# Increased vascularization of the subchondral region in human osteoarthritic femoral head in the elderly

**DOI:** 10.1007/s00418-025-02365-6

**Published:** 2025-03-23

**Authors:** Yuqi He, Katrin Bundkirchen, Shahed Taheri, Ricarda Stauß, Emmanouil Liodakis, Claudia Neunaber, Arndt F. Schilling, Christian Mühlfeld, Stephan Sehmisch, Tilman Graulich

**Affiliations:** 1 Department of Trauma Surgery, Hannover Medical School, Carl-Neuberg Str. 1, 30625 Hannover, Germany; 2 Department of Trauma Surgery, Orthopaedics and Plastic Surgery, University Medical Center Goettingen, Robert Koch Straße 40, 37075 Göttingen, Germany; 3 Department of Orthopaedic and Trauma Surgery, University of Oldenburg, Pius Hospital, Oldenburg, Germany; 4 Department of Trauma, Hand and Reconstructive Surgery, Departments and Institutes of Surgery, Saarland University, Homburg, Germany; 5 Hannover Medical School, Institute of Functional and Applied Anatomy, German Center for Lung Research (DZL), Hannover, Germany

**Keywords:** Subchondral vascularization, Osteoarthritis, Human, Femoral head, Stereology

## Abstract

This study aimed to quantitatively analyze subchondral vascularization during the progression of osteoarthritis (OA) in the elderly, particularly regarding the timing of initial blood vessel emergence and when their density peaks. A total of 129 independent areas from 43 human femoral heads, obtained through arthroplasty for OA or hemiarthroplasty for fractures, were analyzed. The femoral heads were grouped by Kellgren-Lawrence (KL) grades: KL 1 (6 heads), KL 2 (14 heads), KL 3 (10 heads), and KL 4 (13 heads), and the Mankin score was assessed. Quantitative measurements of blood vessel length 1 mm below the tidemark, cartilage volume and thickness, chondrocyte volume, ECM volume, subchondral bone volume, and bone marrow volume were performed using stereology and immunohistochemistry. The most substantial increase in the characteristics of blood vessels within the subchondral region began at KL 3 and peaked at KL 4. Blood vessel volume increased from 6.71 ± 5.84 mm^3^ in group KL 1 to 156.21 ± 138.67 mm^3^ in group KL 4 (*p* < 0.001). Blood vessel surface area showed an increase from 14.78 ± 9.89 cm^2^ (group KL 1) to 125.20 ± 93.18 cm^2^ (group KL 4) (*p* < 0.001). Likewise, blood vessel length grew from 27.53 m (IQR 13.70–65.41 m) in group KL 1 to 112.03 ± 76.07 m in group KL 4 (*p* = 0.001). This study offers deeper insights into the role of vascularization in OA pathophysiology, quantifying subchondral blood vessel characteristics in the femoral head across different OA stages.

**Supplementary Information:**

The online version contains supplementary material available at 10.1007/s00418-025-02365-6.

## Introduction

Osteoarthritis (OA) is a prevalent degenerative joint disease characterized by the progressive degradation of articular cartilage, resulting in joint pain, stiffness, and reduced mobility. It poses a significant burden on the elderly population and is a major contributor to pain and disability (Arden and Nevitt 2006; Harlaar et al. 2022; Vos et al. 2012). OA becomes more common with age, with incidence rates of 21.8% and 31.0% in individuals over 60 and 80 years in Germany, respectively (Postler et al. 2018). This condition also places a substantial socioeconomic burden on developed nations, accounting for 1.0–2.5% of the gross domestic product (GDP) in Europe (Hiligsmann et al. 2013).

Subchondral bone consists of the subchondral bone plate (SBP) and subchondral trabecular bone (STB) (Madry et al. 2010). The SBP is dense and intersected by blood vessels and nerves, while the STB undergoes remodeling. In OA, natural microporous channels and subchondral lacunae form, allowing blood vessel and neurovascular invasion (Taheri et al. 2021, 2023). Vascular endothelial growth factor (VEGF) from hypertrophic chondrocytes and osteoblasts promotes this invasion (Hu and Olsen 2016), increasing oxygen pressure and causing chondrocyte apoptosis (Lafont et al. 2008). Lacunae also permit osteoclast precursor invasion, further degrading cartilage (Zhu et al. 2021).

Several factors like mechanical stress, inflammation, and trauma are known to accelerate the natural progression of OA. The vascularization is regulated by a balance between proangiogenic and antiangiogenic factors (Mapp and Walsh 2012). Whereas under physiological conditions this balance maintains the avascular nature of the articular cartilage, in OA proangiogenic factors lead to increased blood vessel density and permeability (Pesesse et al. 2011). Although recent studies have linked increased osteochondral vascularization with greater cartilage damage and disease severity, particularly in subchondral regions (Walsh et al. 2007; Imhof et al. 1999), they primarily focus on qualitative blood vessel changes. Our study aims to provide a more comprehensive quantitative analysis of vascular morphological changes in the human femoral head, specifically in different stages of OA.

## Materials and methods

### Patient recruitment and ethics statement

The study was conducted according to the principles of the Helsinki Declaration and was approved by the Ethics Committee of Hannover Medical School (Approval number 3377-2016). Participation was entirely voluntary, with patients providing written informed consent before inclusion in the study.

Between January 2022 and February 2023, 43 femoral heads were collected from patients at Hannover Medical School. Inclusion criteria were (1) patients with hip OA undergoing total hip arthroplasty because of severe symptoms, failed conservative treatment, and mobility issues; and (2) elderly patients (> 70 years of age) with displaced femoral neck fractures (Garden III or IV) considered for bipolar hip arthroplasty. Patients with bone or other organ tumors were excluded.

After the inclusion of the femoral heads, they were grouped on the basis of the results of preoperative X-ray examination using the Kellgren-Lawrence (KL) grading system; independent grading was performed by two trained orthopaedic surgeons. Accordingly, the patients and their femoral heads (*n* = 43) were divided into four groups: KL 1 (*n* = 6), KL 2 (*n* = 14), KL 3 (*n* = 10), and KL 4 (*n* = 13), i.e., patients with KL scores of 1, 2, 3, and 4 respectively.

### Preparation of femoral head cubes

The femoral heads were removed during surgery, fixed in formalin at 4 °C for 24 h, and then stored in 70% ethanol (Th. Geyer GmbH & Co. KG, Renningen, Germany) at 4 °C. Their volume was measured via water displacement. Marked with 1-cm parallel lines, the heads were cut with a surgical oscillating saw (see Fig. S1). Cartilage volume was assessed using the Cavalieri method. Fan-shaped sections were created, preserving a 1-cm outer cartilage circumference. Cubes were cut from 1 cm below the cartilage, totalling 1 cm^3^ in volume. A systematic uniform random sampling (SURS) selected three cubes from every third counted cube (Boyce et al. 2010), resulting in 129 cubes stored in 70% ethanol.

### Embedding femoral head samples

Biopsies were embedded natively in an undecalcified state using Technovit 9100 New® (Heraeus Kulzer GmbH, Wehrheim, Germany). Briefly the samples from the femoral head as described in Sec. “Preparation of the femoral head cubes” underwent dehydration in ethanol and isopropanol series (DVH Chemie-Vertrieb GmbH & Co Hannover KG, Hannover, Germany), xylene incubation (J.T.Baker, Deventer, Netherlands), pre-infiltration, and infiltration before being embedded in Technovit 9100 New® using a polymerization solution (Heraeus Kulzer GmbH, Wehrheim, Germany) (Bernhards et al. 1992). Then the cubes were sliced into 5-µm sections using a microtome.

### Histology staining

Hematoxylin and eosin (HE) staining (Carl Roth GmbH, Karlsruhe, Germany) and Safranin O staining were conducted according to Cardiff et al. (2014) and Rosenberg (1971). Immunohistochemistry (IHC) staining was performed according to Magaki et al. (2019). Tissue slides underwent MEA immersion (Merck, Darmstadt, Germany), decalcification, ethanol exposure, Tris-buffered saline rinsing (Sigma-Aldrich Chemie GmbH, Munich, Germany), EDTA incubation (Merck, Darmstadt, Germany), and hydrogen peroxide quenching (Abcam GmbH, Berlin, Germany). IHC staining was performed using the ZytoChem Plus (HRP) Polymer Kit (Zytomed Systems GmbH, Berlin, Germany), involving blocking for 10 min, ready-to-use CD34 antibody (#IR63261-2, Agilent, Santa Clara, USA) application for 1 h, post-block solution (ZytoChem Plus (HRP) Polymer) was added for 20 min, and visualization using ZytoChem Plus (HRP) Polymer (POLHRP-006, Zytomed Systems GmbH, Berlin, Germany) at 20 ℃ for 30 min was performed. TBS was used as the negative control. Although several known antibodies like CD31 or VEGF are well known for blood vessel staining we decided to use the antibody against CD34 as this was able to mark blood vessels in the decalcified tissue. Limitations are potential inaccuracy due to cross staining of endothelial cells and hematopoetic cells.

Counterstaining was done with Mayer’s hemalum solution (Merck, Darmstadt, Germany) and TBS bluing (Sigma-Aldrich Chemie GmbH, Munich, Germany), followed by ethanol and xylene (J.T.Baker, Deventer, Netherlands) treatments for slide preparation. Finally, the slides were mounted with Vitro-Clud embedding glue (R. Langenbrinck GmbH, Emmendingen, Germany).

### Evaluation of degenerative cartilage using the Mankin score

This grading system comprises four parts: structure integrity, cellular abnormalities, matrix staining, and tidemark integrity. The scores for all four features are added to obtain a total Mankin score, which can range from 0 (normal cartilage) to 14 (severe OA) (Pauli et al. 2012; van der Sluijs, et al. 1992). In the present study, all 129 slides stained with Safranin O/Fast Green were evaluated using the Mankin score.

### Measurement and calculation of volumes of tissues and cells

#### Femoral head volume

First, the femoral head was freed from the extra tissue and rest of the femoral neck. Total femoral head volume was obtained using Archimedes’ principle using water displacement. The weight change was recorded which corresponded to the femoral head volume (V(FH)) (Scherle 1970).

#### Cartilage volume

A grid with 200 evenly spaced points (0.5 cm) was used to assess volume density. Points hitting the femoral head and cartilage were counted. The cartilage volume density (P(cartilage)) was calculated and multiplied by the femoral head volume to afford the total cartilage volume (V(cartilage)).

1 $${\mathrm{V}}\left( {{\mathrm{cartilage}}} \right) = {\mathrm{V}}\left( {{\mathrm{FH}}} \right) \times \frac{{{\mathrm{P}}\left( {{\mathrm{cartilage}}} \right)}}{{{\mathrm{P}}\left( {{\mathrm{FH}}} \right)}}$$

#### Subchondral region volume

The volume of the femoral head without cartilage was calculated by subtracting the cartilage volume from the total femoral head volume. The sphere volume formula was used to determine the radius of the femoral head without cartilage (R(FH)). After 1 mm was subtracted from the radius, the inner femoral head volume was recalculated. The subchondral region volume 1 mm below the tidemark (V(SB 1mm)) was obtained by subtracting the inner femoral head volume from the total.

2 $${\mathrm{R}}\left( {{\mathrm{FH}}} \right) = \sqrt[3]{{\frac{{4\pi }}{{{\mathrm{3}}\left[ {{\mathrm{V}}({\mathrm{FH}}){\text{ - V}}\left( {{\mathrm{cartilage}}} \right)} \right]}}}}$$

3 $${\mathrm{V}}\left( {{\text{SB }}1{\mathrm{mm}}} \right) = {\text{ V}}({\mathrm{FH}}){\text{ - V}}({\mathrm{cartilage}}){\text{ - }}\frac{{\mathrm{3}}}{{\mathrm{4}}}\pi \left( {{\mathrm{R}}\left( {{\mathrm{FH}}} \right){\text{ - 1~mm}}} \right)^{3} {\text{ }}$$

#### Cartilage thickness

HE-stained femoral head slides were imaged using the VHX microscope (Keyence GmbH, Neu-Isenburg, Germany). The “2 Points” tool in the VHX software was used to select the top and bottom of the cartilage, automatically calculating the thickness. This was performed for three regions (left, middle, right) of each femoral head slice, and the average cartilage thickness for each sample was reported.

#### Chondrocyte and ECM volumes

The volume densities and total volumes of chondrocytes and extracellular matrix (ECM) were calculated after measuring cartilage thickness. In the VHX software, “Scale” mode was switched to “Mesh” mode with a 100 μm × 100 μm grid. Asterisks at grid intersections, including those on chondrocytes, were counted. The volume densities and total volumes of chondrocytes (V_v_ and V(chondrocyte, cartilage)) and ECM (V_v_ and V(ECM, cartilage)) in cartilage were then calculated using standard formulae.

4 $$\begin{array}{*{20}c} {{\mathrm{V}}_{{\mathrm{V}}} \left( {{\mathrm{chondrocyte}}/{\mathrm{cartilage}}} \right) = \frac{{{\mathrm{P}}\left( {{\mathrm{chondrocyte}}} \right)}}{{{\mathrm{P}}\left( {{\mathrm{cartilage}}} \right)}}} \\ \end{array}$$

5 $${\mathrm{V}}\left( {{\mathrm{chondrocyte}},{\text{ cartilage}}} \right) = {\mathrm{V}}\left( {{\mathrm{cartilage}}} \right) \times {\mathrm{V}}_{{\mathrm{V}}} \left( {\frac{{{\mathrm{chondrocyte}}}}{{{\mathrm{cartilage}}}}} \right)$$

6 $$\begin{array}{*{20}c} {{\mathrm{V}}_{{\mathrm{V}}} \left( {{\mathrm{ECM}}/{\mathrm{cartilage}}} \right) = \frac{{{\mathrm{P}}\left( {{\mathrm{cartilage}}} \right){ } - {\text{ P}}\left( {{\mathrm{chondrocyte}}} \right)}}{{{\mathrm{P}}\left( {{\mathrm{cartilage}}} \right)}}} \\ \end{array}$$

7 $${\mathrm{V}}\left( {{\mathrm{ECM}},{\text{ cartilage}}} \right) = {\mathrm{V}}\left( {{\mathrm{cartilage}}} \right) \times {\mathrm{V}}_{{\mathrm{V}}} \left( {{\mathrm{ECM}}/{\mathrm{cartilage}}} \right)$$

#### Subchondral bone and bone marrow volumes

The point counting method was used to calculate volume densities and total volumes of subchondral bone and bone marrow. Subchondral bone volume was measured at 1 mm below the tidemark using ×40 magnification. A 500 μm × 500 μm grid with 160 intersection points was applied to cartilage images, and points intersecting trabecular bone were counted. Volume densities and total volumes for subchondral bone (V_v_ and V (SB/SR)) and bone marrow (V_v_ and V (BM/SR)) were calculated using standard formulae.

8 $$\begin{array}{*{20}c} {{\mathrm{V}}_{{\mathrm{V}}} \left( {\frac{{{\mathrm{SB}}}}{{{\mathrm{SR}}}}} \right) = \frac{{{\mathrm{P}}\left( {{\mathrm{SB}}} \right)}}{{{\mathrm{P}}\left( {{\mathrm{SR}}} \right)}}} \\ \end{array}$$

9 $${{\mathrm{V}}\left( {{\mathrm{SB}},{\text{ SR}}} \right) = {\mathrm{V}}\left( {{\mathrm{SR}}} \right) \times {\mathrm{V}}_{{\mathrm{V}}} }$$

10 $$\begin{array}{*{20}c} {{\mathrm{V}}_{{\mathrm{V}}} \left( {{\mathrm{BM}}/{\mathrm{SR}}} \right) = \frac{{{\mathrm{P}}\left( {{\mathrm{SR}}} \right){ } - {\text{ P}}\left( {{\mathrm{SB}}} \right)}}{{{\mathrm{P}}\left( {{\mathrm{SR}}} \right)}}} \\ \end{array}$$

11 $${\mathrm{V}}\left( {{\mathrm{BM}},{\text{ SR}}} \right) = {\mathrm{V}}\left( {{\mathrm{SR}}} \right) \times {\mathrm{V}}_{{\mathrm{V}}} \left( {{\mathrm{BM}}/{\mathrm{SR}}} \right){\text{ }}$$

#### Blood vessel volume

At ×100 magnification, the subchondral region 1 mm below the tidemark was selected using the VHX software’s “Rectangle” tool to calculate its area (A(SR 1mm)). The “Free Line” tool outlined the blood vessels, and the software determined their area (A(vessels)). The volume density of blood vessels (V_v_(vessels/SR 1mm)) was calculated by dividing the area of blood vessels by the area of the subchondral region. The total volume of blood vessels (V(vessels, SR 1mm)) was obtained by multiplying the volume density of blood vessels by the volume of the subchondral region.

12 $$\begin{array}{*{20}c} {{\mathrm{V}}_{{\mathrm{V}}} \left( {{\mathrm{vessels}}/{\text{SR }}1{\mathrm{mm}}} \right) = \frac{{{\mathrm{A}}\left( {{\mathrm{vessels}}} \right)}}{{{\mathrm{A}}\left( {{\text{SR }}1{\mathrm{mm}}} \right)}} } \\ \end{array}$$

13 $${\mathrm{V}}\left( {{\mathrm{vessels}},{\text{ SR }}1{\mathrm{mm}}} \right) = {\mathrm{V}}\left( {{\text{SR }}1{\mathrm{mm}}} \right) \times {\mathrm{V}}_{{\mathrm{V}}} \left( {{\mathrm{vessels}}/{\text{SR }}1{\mathrm{mm}}} \right)$$

#### Blood vessel surface area

Femoral head images were overlaid with a transparent grid of 34 cycloids aligned vertically to the articular surface. The numbers of intersections (I) with blood vessel boundaries, left endpoints (N), and total cycloid length (LC) were calculated. The surface area density of blood vessels (S_v_(vessels/SR 1mm)) was computed, and the total blood vessel surface area in the subchondral region 1 mm below the tidemark was determined by multiplying the surface area density by the subchondral region volume.

14 $$\begin{array}{*{20}c} {{\mathrm{S}}_{{\mathrm{V}}} \left( {{\mathrm{vessels}}/{\text{SR }}1{\mathrm{mm}}} \right) = \frac{{2{\mathrm{I}}}}{{\text{N LC}}} } \\ \end{array}$$

15 $${\mathrm{S}}\left( {{\mathrm{vessels}},{\text{ SR }}1{\mathrm{mm}}} \right) = {\mathrm{V}}\left( {{\text{SR }}1{\mathrm{mm}}} \right) \times {\mathrm{S}}_{{\mathrm{V}}} \left( {\frac{{{\mathrm{vessels}}}}{{{\mathrm{SR}}}}1{\mathrm{mm}}} \right){\text{ }}$$

#### Blood vessel length

To calculate blood vessel length, we assumed the blood vessel to be a long and perfect cylinder. By combining the cylinder volume formula with surface area formula, the formulae for calculating the length density (L_V_(vessels/SR 1mm)) and total length (L(vessels, SR 1mm)) of the blood vessels were derived as follows:

16 $$\begin{array}{*{20}c} {{\mathrm{L}}_{{\mathrm{V}}} \left( {{\mathrm{vessels}}/{\text{SR }}1{\mathrm{mm}}} \right) = \left( {\frac{1}{{4{\uppi }}}} \right)\left( {\frac{{{\mathrm{S}}_{{\mathrm{V}}} \left( {{\mathrm{vessels}}/{\text{SR }}1{\mathrm{mm}}} \right)^{2} }}{{{\mathrm{V}}_{{\mathrm{V}}} \left( {{\mathrm{vessels}}/{\text{SR }}1{\mathrm{mm}}} \right)}}} \right)} \\ \end{array}$$

17 $$L\left( {{\mathrm{vessels}},{\text{ SR }}1{\mathrm{mm}}} \right) = {\mathrm{V}}\left( {{\text{SR }}1{\mathrm{mm}}} \right) \times {\mathrm{L}}_{{\mathrm{V}}} \left( {{\mathrm{vessels}}/{\text{SR }}1{\mathrm{mm}}} \right)$$

### Micro CT

Four cartilage-bone biopsies from different KL grades underwent microCT imaging. Previously examined histologically, the goal was to obtain high-resolution scans and overlay the microCT images with their histological counterparts. The biopsies, embedded in polymethyl methacrylate (PMMA) blocks, were positioned in the sample holder for microCT scanning.

The microCT scans were performed utilizing a high-definition micro-CT system (µCT 50, SCANCO Medical AG, Switzerland) with specific imaging parameters. The scan settings included an energy level of 90 kVp, intensity set at 155 µA, voxel size of 7.4 µm, and an integration time of 1000 ms. The subsequent segmentation of the acquired images was accomplished using Scanco’s OpenVMS script, applying standard threshold settings for image segmentation.

### Statistical analysis

Statistical analyses and image creation were conducted using GraphPad Prism 9 (v.9.3.1, GraphPad Company, Boston, USA). Data normality was assessed with the Shapiro–Wilk test. Normally distributed data were expressed as mean ± standard deviation and analyzed using one-way analysis of variance (ANOVA), while non-normally distributed data were presented as median and interquartile range (IQR) and analyzed with the Kruskal–Wallis* H* test. Spearman’s rank correlation and linear regression assessed relationships between cartilage, subchondral bone, and blood vessels. Rank analysis of covariance (ANCOVA) and partial correlation were used to control femoral head volume confounding (Quade 1967). A *p* value ≤ 0.05 indicated statistical significance.

## Results

### General characteristics of patients and femoral heads

In total, 43 patients and femoral heads were included in this study, with 26 women and 17 men and an average age of 76.84 ± 12.85 years. Table 1 presents the age and sex ratios of the patients in each group. No significant differences were observed in age and sex across the four groups (*p*_age_ = 0.359, *p*_sex_ = 0.136). Among the enrolled patients, 23 underwent bipolar hip arthroplasty owing to a hip neck fracture and 20 underwent total hip arthroplasty for OA.

**Table 1:** General characteristics of patients and femoral heads in the four groups

Group	KL 1	KL 2	KL 3	KL 4	Total	*p*
*n*	6	14	10	13	43	–
Age (years)	82.00 ± 12.76	74.86 ± 10.86	81.00 ± 12.33	73.38 ± 14.94	76.84 ± 12.85	0.359
Sex						
Female	4	9	3	10	26	0.136
Male	2	5	7	3	17	
Reference volume						
Femoral head (cm^3^)	47.64 ± 8.77	53.50 ± 17.64	54.64 ± 12.89	56.92 ± 27.34	53.90 ± 18.63	0.814
Cartilage (cm^3^)	13.55 ± 2.69 a	15.74 ± 3.65 a	15.23 ± 4.07 a	11.59 ± 3.43 b	14.06 ± 3.89	0.024
Subchondral region below the tidemark 1 mm (cm^3^)	4.62 (IQR 4.44–5.14)	5.20 ± 1.31	5.31 ± 0.88	5.62 ± 1.99	4.98 (IQR 4.38–5.96)	0.084

Values with letter a and b are significantly different across groups (*p* < 0.05)

The total volumes of the femoral heads, cartilage, and subchondral region were measured as reference volumes. The overall femoral head volume was 53.90 ± 18.63 cm^3^, with no significant differences between the groups (*p* = 0.814). The cartilage volume was 14.06 ± 3.89 cm^3^, with group KL 4 showing significantly lower cartilage volume (*p* = 0.024). The subchondral region volume 1 mm below the tidemark was 4.98 cm^3^ (IQR 4.38–5.96 cm^3^), with no significant differences between groups (*p* = 0.835). Detailed values are shown in Table 1.

### Stereological analysis of blood vessel changes in subchondral region

**Fig. 1:**
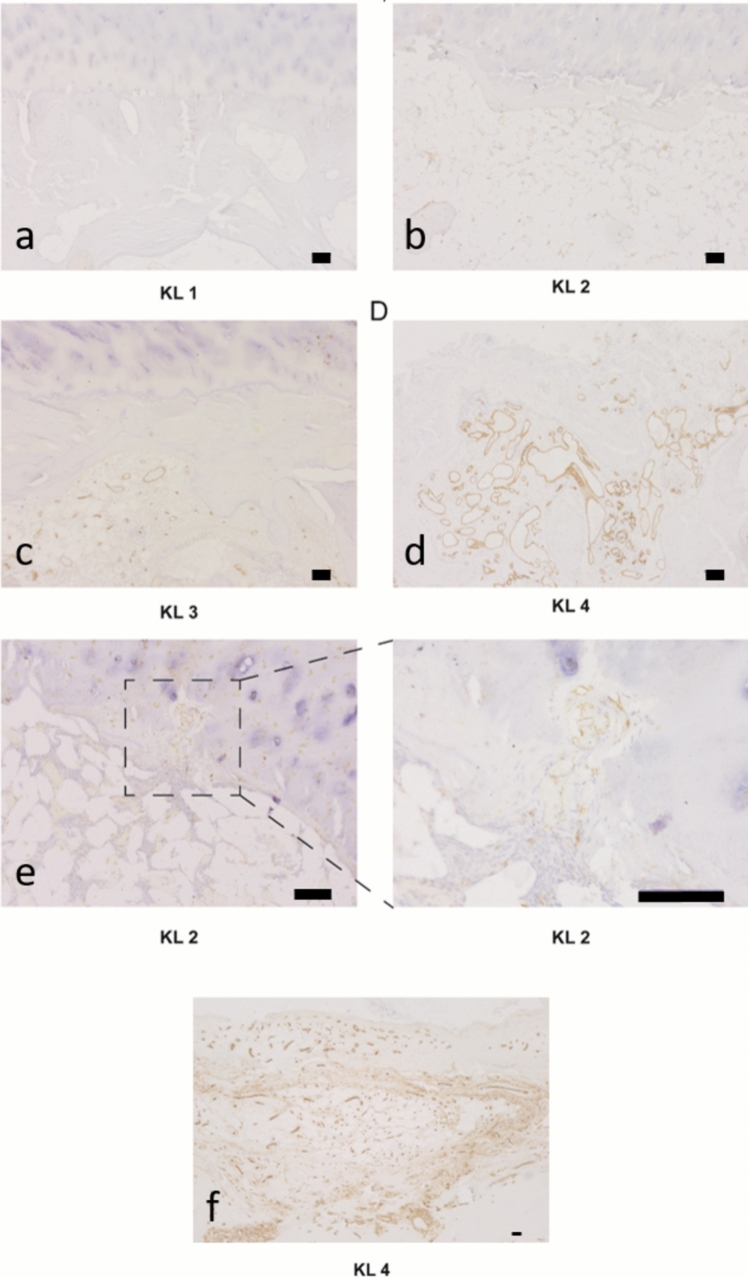
Morphological changes of vessels in the subchondral region. **A**–**F** IHC staining was performed using the CD34 antibody, a recognized marker specific for vascular endothelial cells. The CD34-positive cells are represented by distinct brown circular staining. Group KL 1 (**A**) and group KL 2 (**B**) showed rare CD34-positive cells. Group KL 3 (**C**) exhibited a moderate presence of CD34-positive cells in the vicinity of the tidemark. Group KL 4 (**D**) displayed an abundance of CD34-positive cells below the tidemark. At the eatly stage, some vessels were observed crossing the tidemark (**E**), while at the late stage, extensive vascularization occupied the cartilage (**F**). Scale bar 100 μm.* N*_patients_ = 43 (total femoral heads),* n*_biopsies_ = 129 (3 biopsies/femoral head)

In the subchondral region 1 mm below the tidemark, groups KL 1 (Fig. 1A) and KL 2 (Fig. 1B) showed sparse or absent blood vessels, mainly located away from the tidemark. Group KL 3 (Fig. 1C) had moderate vascularity near the tidemark, while group KL 4 (Fig. 1D) exhibited an extensive blood vessel network concentrated near it. Blood vessels began crossing the tidemark into the cartilage in KL 2 (Fig. 1E), and in advanced stages of degeneration, widespread vascular proliferation obscured normal chondrocytes and ECM (Fig. 1F).

**Table 2:** Vessel parameters in the four groups

Group	KL 1	KL 2	KL 3	KL 4	Total	*p*
Vessel crossing the tidemark						
*n*	0	8	12	15	–	–
Density (/cm)	0	0.18	0.40	0.36	–	–
Vessel volume						
V_v_ (vessel/subchondral region) (%)	0.06 (IQR 0–0.19)*#	0.70 (IQR 0–1.21)*#	0.72 (IQR 0.08–1.77)*	2.24 (IQR 0.92–2.85)	0.26 (IQR 0.04–1.85)	< 0.001
V (vessel, subchondral region) (cm^3^)	6.71 ± 5.84*#	7.82 (IQR 0.89–14.58)*#	57.14 ± 42.68*	156.21 ± 138.67	27.64 (IQR 5.16–90.05)	< 0.001
Vessel surface area						
S_v_ (vessel/subchondral region) (/cm)	1.13 (IQR 0–5.29)*#	2.93 (IQR 0–6.36)*#	14.13 (IQR 3.11–20.83)	15.95 (IQR 12.54–21.29)	6.68 (IQR 1.41–17.56)	< 0.001
S (vessel, subchondral region) (cm^2^)	14.78 ± 9.90*#	18.00 (IQR 6.42–33.78)*#	76.41 ± 49.44	125.20 ± 93.18	37.48 (IQR 15.15–92.81)	< 0.001
Vessel length						
Lv (vessel/subchondral region) (/cm^2^)	205.45 (IQR 0–1323.59)*#	652.26 (IQR 0–1714.88)*#	1657.39 (IQR 602.26–4230.45)	1313.94 (IQR 671.86–2256.58)	966.03 (IQR 216.06–2133.60)	0.001
L (vessel, subchondral region) (m)	27.53 (IQR 13.70–65.41)*#	50.78 ± 33.80*#	121.89 (IQR 43.23–217.42)	112.03 ± 76.07	61.52 (IQR 36.11–119.62)	0.001

*p < 0.05 vs. group KL 4; #*p* < 0.05 vs. group KL 3

Blood vessel characteristics were analyzed across OA severity groups (KL 1–4). Blood vessel volume in the subchondral region 1 mm below the tidemark increased with OA progression, peaking in group KL 4 (156.21 ± 138.67 mm^3^) and significantly higher than groups KL 1 and KL 2 (*p* < 0.001) and KL 3 (*p* = 0.010). Group KL 3 followed with 57.14 ± 42.68 mm^3^, significantly higher than KL 1 (*p* = 0.024) and KL 2 (*p* = 0.009).

Blood vessel surface area also increased with OA severity, with groups KL 3 and KL 4 showing comparable areas (76.41 ± 49.44 cm^2^ and 125.20 ± 93.18 cm^2^), both significantly higher than KL 1 (14.78 ± 9.90 cm^2^) and KL 2 (18.01 cm^2^, *p* < 0.001). Blood vessel length followed a similar trend, with group KL 3 measuring 121.89 m (IQR 43.23–217.42 m) and KL 4 at 112.03 ± 76.07 m, both significantly longer than KL 1 and KL 2 (*p* = 0.728). Positive correlations were also found between Mankin scores and blood vessel densities. Table 2 summarizes these values.

### Results from Micro CT

**Fig. 2:**
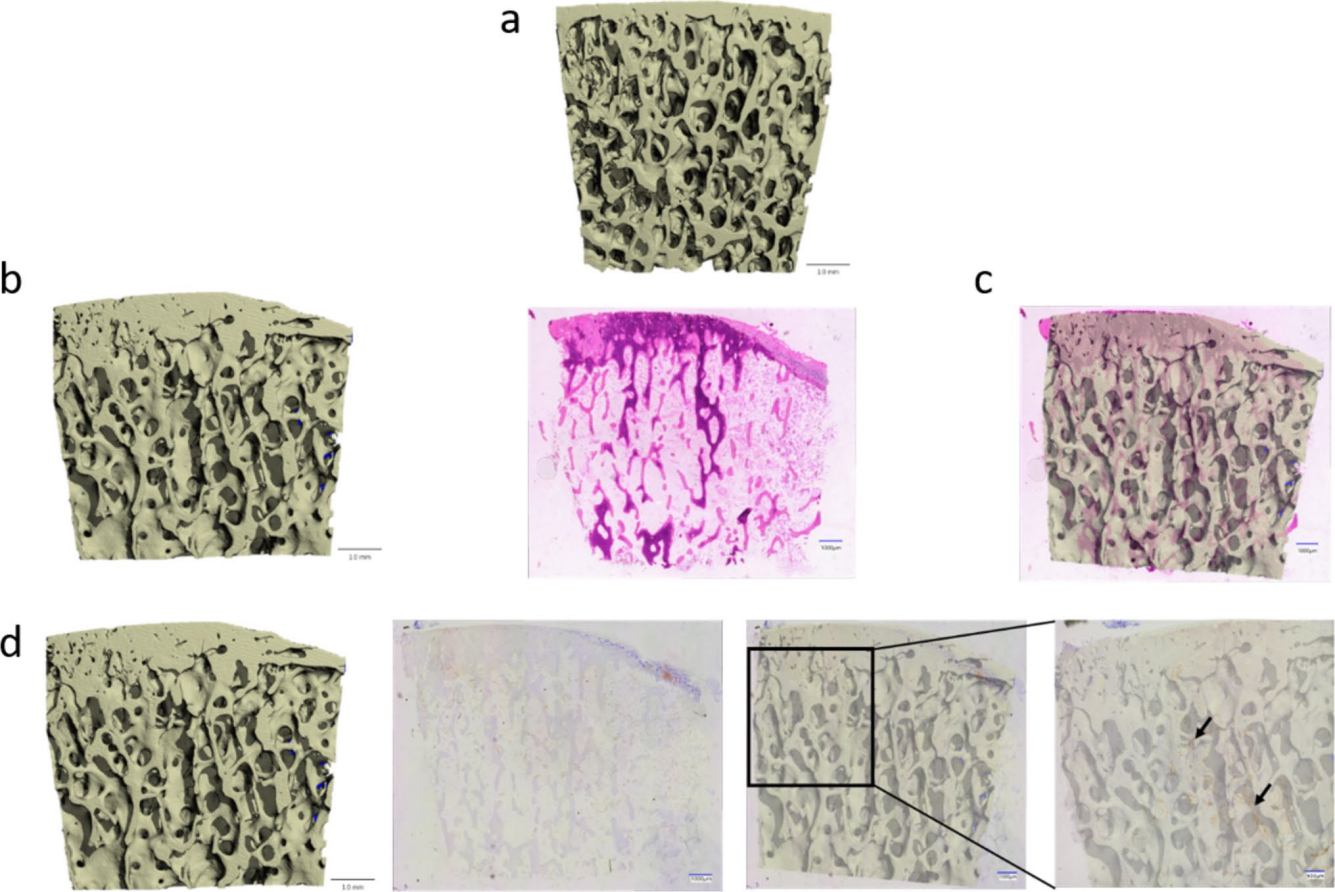
3D-reconstructed bone model and histological image. **A** 3D-reconstructed bone model. **B** The comparison between microCT findings and HE staining of a KL 4 biopsy reveals subchondral bone sclerosis and cartilage erosion on the left side, contrasting with a healthier bone and cartilage structure on the right, accompanied by pronounced accumulation of mineralized matrix and osteoids on the end-stage side. **C** Merge picture from Fig. 2B. **D** The 3D-reconstructed bone model undergoes IHC staining for CD34, revealing an increased accumulation of vascular structures on the end-stage side, suggesting a potential link between vascularization patterns and bone/cartilage structural changes

3D-reconstructed bone model offered a comprehensive view of the upper surface of the PMMA block housing the cartilage-bone biopsy (Fig. 2A). This 3D representation allows for an in-depth visualization of the overall specimen, aiding in the spatial assessment of the structural integrity of both bone and cartilage. The microCT and HE pictures were compared from a KL 4 biopsy. This biopsy reveals distinct pathological features, including subchondral bone sclerosis and complete cartilage erosion on the left side, in contrast to a comparatively healthier bone and cartilage structure on the right. A pronounced accumulation of mineralized matrix and osteoids, visible as dark-purple staining, is evident on the end-stage side (Fig. 2B).

Overlay the 3D-reconstructed bone model from the microCT with the corresponding HE-stained image, thereby providing a precise correlation between the structural details revealed by these two complementary techniques. This overlay highlights the spatial relationship between the microCT imaging and histological features, improving the interpretation of the observed structural changes (Fig. 2C). Meanwhile, merge the 3D-reconstructed bone model and CD34-stained image to highlight endothelial cells. Increased vascular structures were observed on the end-stage side of the biopsy (Fig. 2D). In OA the dense subchondral bone sclerosis can be observed in 3D-reconstructed bone model from the microCT with the corresponding HE-stained image. Furthermore, in the non-calcified subchondral bone areas increased vascular invasion is observed in OA. Whereas in healthy cartilage the HE staining reveals a blue dense tidemark, in OA the calcified subchondral bone shows in both the HE staining and the 3D-reconstructed bone model, the condensation of bone material by a rich blue and red staining of the HE and a thickening of the matrix in the 3D-reconstructed bone model.

### Correlations of blood vessel values with cartilage and subchondral bone in OA

Total blood vessel volume showed strong negative correlations with cartilage volume, thickness (*p* < 0.001), chondrocyte volume (*p* = 0.006), ECM volume (*p* < 0.001), and bone marrow volume (*p* = 0.038), and a moderate positive correlation with subchondral bone volume (*p* < 0.001). Blood vessel surface area had moderate negative correlations with total cartilage volume (*p* < 0.001), cartilage thickness (*p* = 0.002), chondrocyte volume (*p* = 0.015), and ECM volume (*p* = 0.004), and a moderate positive correlation with subchondral bone volume (*p* = 0.003). Blood vessel length also showed moderate negative correlations with cartilage (*p* = 0.036) and chondrocyte volumes (*p* = 0.014, Table 3).

**Table 3:** Correlation of vessel total values with cartilage and subchondral bone

	V (cartilage)	Cartilage thickness	V (chondrocytes/cartilage) (cm^3^)	V (ECM/cartilage) (cm^3^)	V (subchondral bone)	V (bone marrow)
V (vessels, subchondral bone)						
rs	− 0.663	− 0.558	− 0.447	− 0.528	0.538	− 0.317
* p*	< 0.001	< 0.001	0.006	< 0.001	< 0.001	0.038
*N*	43	43	37	37	43	43
S (vessels, subchondral bone)						
rs	− 0.663	− 0.558	− 0.447	− 0.528	0.538	− 0.317
*p*	< 0.001	< 0.001	0.006	< 0.001	< 0.001	0.038
N	43	43	37	37	43	43
L (vessels, subchondral bone)						
rs	− 0.322	–	− 0.401	–		–
* p*	0.036	0.186	0.014	0.075	0.409	0.489
*N*	43	43	37	37	43	43

### Morphological changes in the cartilage

**Fig. 3:**
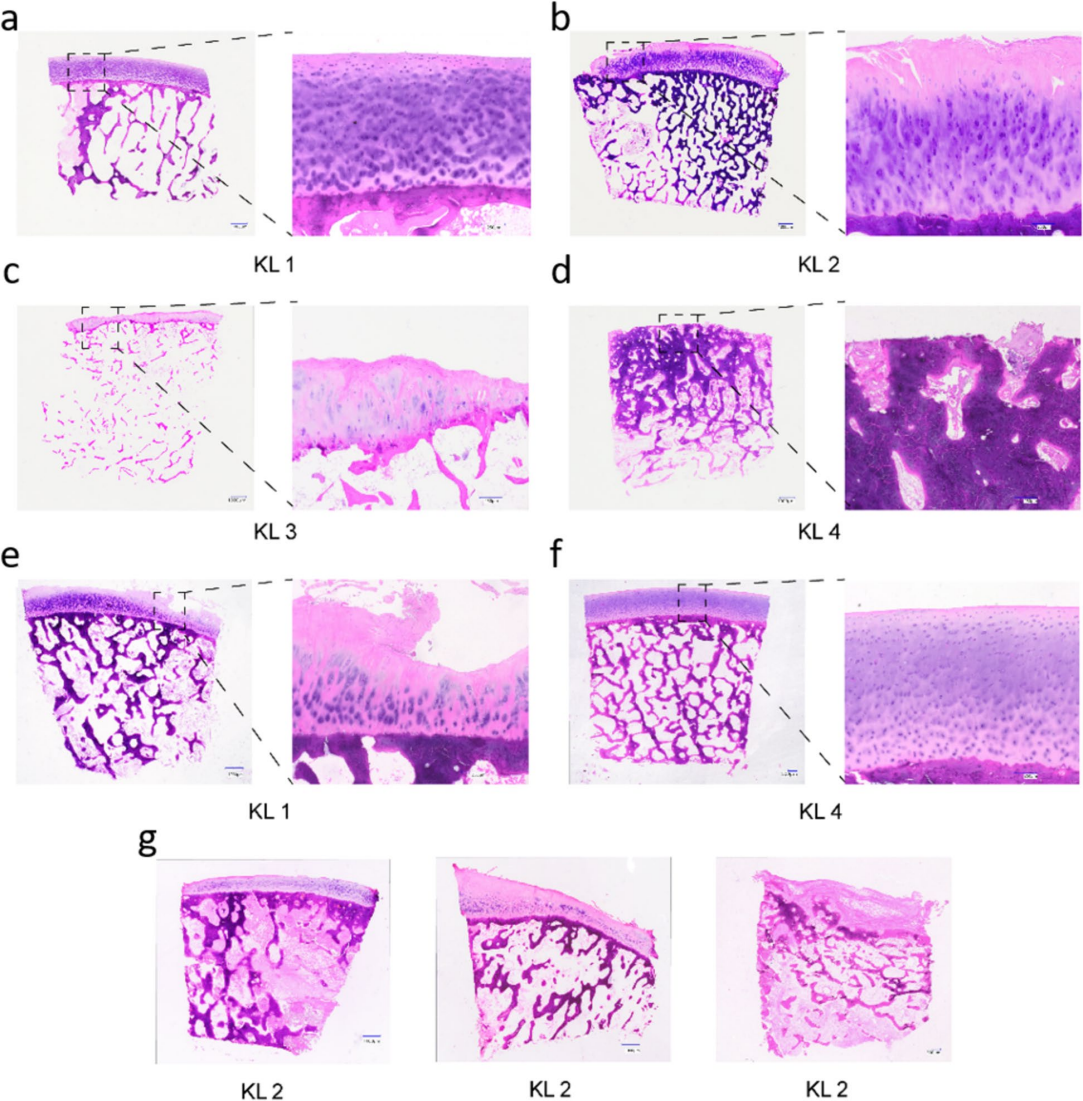
HE staining representing variations in cartilage status. **A** Femoral head in group KL 1: intact and smooth cartilage. Chondrocytes were round or elliptical and regularly arranged. **B** Femoral head in group KL 2: the cartilage begins to degenerate, the surface reveals some cracks and clefts, and chondrocytes exhibit atypical morphology with increasing cell size and irregularity in shape. **C** Femoral head in group KL 3: the cartilage structure experienced further damage, evident by the presence of several deep fissures and cracks on the surface. Chondrocytes increase significantly in cell size and diverge from their characteristic round or elliptical form. **D** Femoral head in group KL 4: evident and complete disappearance of the cartilage. The subchondral bone showed distinct signs of sclerosis, apparent thickening, and disorganized structure. **E** Exception slide for femoral head in group KL 1: the cartilage surface exhibits clear cracks, and chondrocyte shapes become irregular. **F** Exception slide for femoral head in group KL 4: the cartilage is complete, and chondrocytes are in good shape and line up regularly. **G** Three cartilage slides from the same femoral head: the left figure depicts healthy, intact cartilage; the middle figure indicates initial signs of cartilage degeneration, and the right figure represents a severely deteriorated cartilage. The scale bar for the complete femoral head figures is set at 1000 µm, and the enlarged cartilage figures have a scale bar of 250 µm. Sample size:* N*_patients_ = 43 (total femoral heads),* n*_biopsies_ = 129 (3 biopsies/femoral head)

HE staining of 129 biopsies from 43 femoral heads revealed cartilage degeneration corresponding to OA progression, from intact cartilage with healthy chondrocytes in KL 1 to severe fragmentation and subchondral bone sclerosis in KL 4 (Fig. 3). However, some KL 1 biopsies showed unexpected cartilage damage resembling higher KL groups, while certain KL 4 biopsies displayed relatively healthy cartilage, indicating occasional discrepancies between histomorphological images and KL classification.

### Severe OA femoral heads and high Mankin scores

**Fig. 4:**
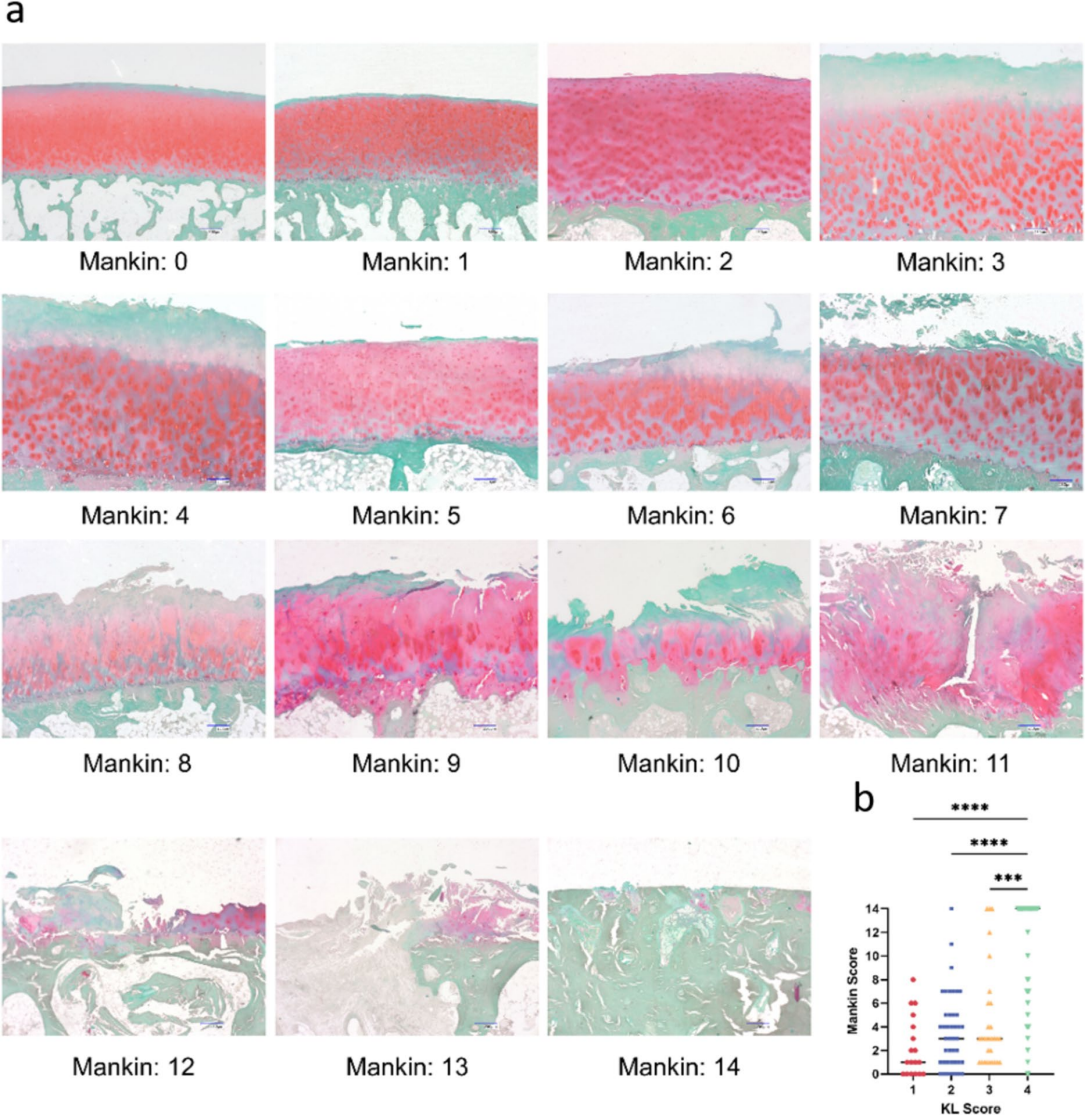
Safranin O/Light green staining showed Mankin scores in different cartilage statuses. With an increase in the Mankin score from 0 to 14, the health of the femoral head significantly deteriorates. Initially, the cartilage surface is smooth, the collagen matrix is stained red and chondrocytes are evenly distributed (Mankin score 0). As the score increases, the cartilage begins to fibrillate, the color of the collagen matrix fades, and chondrocytes start to cluster and proliferate (Mankin score 1–8). With further score increments, the cartilage becomes thinner, the collagen matrix turns white, indicating OA severity (Mankin score 9–12). In the highest scoring stages, the cartilage is severely worn or absent, the collagen matrix becomes disorganized, and the tidemark is blurred (Mankin score 13–14). Scale bar 250 µm. **B** Mankin scores in four KL groups. Group KL 4 exhibits the highest Mankin score compared to the other three groups. Red dots represent the data in group KL 1, blue dots represent group KL 2, yellow dots represent group KL 3, and green dots represent group KL 4. Sample size:* N*_patients_ = 43 (total femoral heads),* n*_biopsies_ = 129 (3 biopsies/femoral head)

A Mankin score of 0 indicated smooth, intact cartilage with regularly spaced chondrocytes and no degeneration. As the score increased from 1 to 14, progressive cartilage degeneration was observed, including fibrillation, chondrocyte clustering, and matrix disorganization. Severe cases showed deep fibrillation, cartilage erosion, and tidemark breaches (Fig. 4). Group KL 4 had the highest Mankin score of 14 (IQR 6–14), significantly higher than the other groups (*p* < 0.001), while no significant differences were found among the other groups (*p* > 0.050).

### Stereological analysis of cartilage and subchondral bone changes

Cartilage thickness significantly decreased across KL groups (*p* < 0.001). While chondrocyte and ECM volume densities showed no significant differences (*p* = 0.283), their total volumes were lowest in KL 4 (chondrocytes: *p* < 0.001, ECM: *p* = 0.002), with similar volumes in KL 1–3 (*p* > 0.050). Subchondral bone in KL 1 appeared normal but showed progressive sclerosis, trabecular coalescence, and cystic changes in advanced OA. The Mankin score was strongly negatively correlated with cartilage thickness (rs = − 0.738,* R*^2^ = 0.667, *p* < 0.001), weakly with chondrocyte and ECM volume densities (rs = − 0.223,* R*^2^ = 0.299, *p* = 0.023), and moderately correlated with subchondral bone (rs = 0.434,* R*^2^ = 0.384, *p* < 0.001) and marrow volume densities (rs = − 0.434,* R*^2^ = 0.384, *p* < 0.001). Full data are in Tables S2, S3, and Fig. S2.

## Discussion

Current research on the molecular and mechanistic processes of OA progression highlights subchondral vascularization as a significant factor in OA development, primarily based on animal and knee studies (Mapp et al. 2008). This study presents a comprehensive analysis of morphological changes in human femoral heads, quantitatively assessing increases in subchondral vascularization during OA progression using histochemical and stereological methods. We found that higher radiological KL grades correlated with more severe cartilage degradation and increased vascularization in the subchondral region. Vascularization occurred prior to cartilage degeneration. This analysis enhances our understanding of OA pathogenesis, providing valuable reference data for both healthy and OA-affected femoral heads and laying the groundwork for future research in this area.

Stereological analysis offers a practical and scientifically robust method for generating accurate quantitative estimates of subtle structural changes in tissues using histological sections (Scherle 1970; Weibel et al. 1966). It extracts 3D quantitative information from 2D images, such as those from microscopy, employing statistical sampling principles and stochastic geometric theory to estimate 3D tissue structures at the whole-organ level. Unlike traditional 2D techniques, stereological methods avoid bias and assumptions owing to their strong statistical foundations. In our previous studies, we effectively estimated key characteristics of cardiac vasculature (Mühlfeld 2014) and axons in the tracheal wall (Graulich et al. 2014) using stereological analysis.

Vascularization plays a crucial role in OA progression (Findlay 2007). In our study, we observed the highest blood vessel volume in group KL 4, followed by KL 3. Surface area and blood vessel length were similar in KL 3 and KL 4, both exceeding those in KL 1 and KL 2. Similar findings have been observed in animal models and knee joints, with increased blood vessel density in OA (Mapp et al. 2008). Blood vessels crossing the tidemark is a remarkable characteristic of OA. Severe OA has more blood vessels crossing the tidemark compared with slight and moderate OA.

We further conducted a correlation analysis to examine the relationship between vascularization and the various OA indicators, including cartilage alterations, chondrocytes, ECM, subchondral bone, and bone marrow. The vascularization changes showed a moderate to strong correlation with cartilage alterations and a moderate correlation with the total volumes of chondrocytes and ECM. Conversely, blood vessel changes exhibited weak to no association with subchondral bone modifications and no relation to the proportion of chondrocytes and ECM.

In a healthy state, blood vessels are crucial for supplying nutrients and oxygen to chondrocytes, maintaining cartilage integrity. However, in OA, neovessels may contribute to cartilage degeneration by altering the subchondral bone environment, affecting oxygen levels, nutrient availability, and growth factor concentrations, which indirectly impacts cartilage health. OA also activates the mechanistic target of rapamycin complex 1 (mTORC1), which plays a critical role in chondrocyte metabolism and disease progression. Increased nutritional support activates mTORC1 in chondrocytes, exacerbating OA development (Pal et al. 2015). Notably, activated mTORC1 promotes the growth of H-type vessels, enhancing nutrient supply to cartilage (Liu et al. 2023; Lu et al. 2018). This creates a positive feedback loop, where mTORC1 activation in chondrocytes influences the formation of subchondral H-type vessels, further impacting OA progression.

In OA progression, subchondral structures undergo changes, including tidemark advancement, blood vessel traversal, and calcified cartilage thickening. Our study observed the parallels in the evolution of the volume of cartilage and subchondral bone as the KL scores increase. This may suggest that cartilage and subchondral bone disturbances are modest in early and mid-stage OA, only becoming severe in late-stage OA. This observation indicates that besides chondrocytes, disorders of subchondral bone may also contribute to OA.

In mid-stage OA (KL 3), blood vessel growth begins without significant cartilage volume loss. By late stage (KL 4), vascularization becomes more pronounced alongside severe cartilage degradation. This suggests that angiogenesis may precede and contribute to cartilage deterioration in advanced OA. Disruption of the cartilage environment by new subchondral blood vessels could accelerate OA progression, highlighting the potential for targeting vascularization in early OA interventions.

Tidemark advancement and blood vessel traversal, first noted in KL 2, increased through KL 3 and KL 4, although quantification in KL 4 was limited as a result of cartilage loss. Blood vessels crossing the tidemark, a known OA indicator in animal models and human knees, were extended to the human hip in this study. Calcified cartilage thickening, marked by microcracks, edema, and bleeding in the subchondral region, was observed, with eburnation exposing calcified cartilage in severe cases. In KL 4, calcified cartilage multiplication, increased thickness, and higher bone density were prominent, driven by active bone cell and chondrocyte proliferation and calcium deposition (Madry et al. 2010),

As OA progresses and cartilage degeneration is initiated, the necessity for nutrients and oxygen in the cartilage increases. Consequently, blood vessels extend towards the cartilage, for nutrients and waste removal (Hugle and Geurts 2017). These precise processes and mechanisms are complex and not yet fully understood. In early OA “tidemark duplication” is observed. In this process, an additional tidemark is formed, increasing the thickness of the calcified cartilage and as OA progresses a breakdown of the tidemark’s barrier function is observed. This alteration results in the invasion of the blood vessels, inflammation, and further cartilage degradation (Goldring and Goldring 2010). The increased expression of proteins like Runx2, BMPs, and alkaline phosphatase (Rees and Ali 1988) that promotes calcification has been suggested as a possible mechanism. These calcification processes may also be triggered by chondrocytes and can stimulate bone remodeling (Burr and Gallant 2012). Another theory proposes that tidemark breakdown is caused by inflammatory mediators that contribute to the tidemark disruption and movement into the cartilage (Lyons et al. 2005).

This study utilized stereological methods to quantify structures in a reference area of the femoral head, assuming an idealized spherical shape for calculations (Foldager et al. 2015; Kamp et al. 2011). In general this method has limitations due to the sampling method with a potential bias. In particular this approach has limited generalizability although the data remained internally comparable. Only three biopsies per head were sampled because of the femoral head’s size and varying OA degrees, which may restrict representation of the full OA landscape and affect the accuracy of structural density measurements. Furthermore, although radiological data suggest that these morphological changes might be at least observed at the knee joint they are not generalizable. Small, non-weight-bearing joints, in particular, could show other morphological changes. Although CD34 was used to visualize blood vessels (Sidney et al. 2014), it cannot differentiate between new and mature blood vessels (Fina et al. 1990). Future studies should include specific neovascularization biomarkers, such as CD31 (Kim et al. 2010) or vascular endothelial growth factor (Murukesh et al. 2010), to better investigate vasculogenesis and angiogenesis in OA. Moreover, vascularization often coincides with neural growth (Eichmann and Thomas 2013). However, current research on neurogenesis in the context of OA remains scarce. Therefore, further exploration in this research area could fill this literature gap.

## Conclusion

This study quantitatively analyzed the volume, surface area, and length of blood vessels located 1 cm beneath the subchondral layer of the femoral head in elderly patients with OA and examined the vascular morphological characteristics across different OA stages. The results showed that the blood vessel volume was highest in late-stage OA, followed by the mid-late stage. The surface area and length of the blood vessels in the mid-late and late stages were similar, both exceeding those in the early and mid-stages. Additionally, a correlation between vascularization and cartilage degradation was identified, with vascularization preceding the onset of cartilage degeneration. This temporal relationship suggests that vascularization may contribute to the initiation of cartilage degradation and the early pathogenesis of OA.

## Supplementary Information

Below is the link to the electronic supplementary material.

Supplementary file1 (DOCX 1975 KB)

## Data Availability

No datasets were generated or analysed during the current study.
