# Supplementary material for: Increased vascularization of the subchondral region in human osteoarthritic femoral head in the elderly
Source: Histochem Cell Biol. 2025 Mar 23;163(1):39. doi: 10.1007/s00418-025-02365-6 (PMC11930877; doi:10.1007/s00418-025-02365-6)
Supplement: Supplementary file 1 — Supplementary file1 (DOCX 1975 KB) [file 418_2025_2365_MOESM1_ESM.docx]

**Increased vascularization of the subchondral region in human osteoarthritic femoral head in the elderly.**

***Running title: A quantitative analysis using stereology***

Histochemistry and Cell Biology

Yuqi He, MD; Katrin Bundkirchen, Dr. vet med.; Shahed Taheri, PhD; Ricarda Stauß, Dr. med.; Emmanouil Liodakis, Prof. Dr. med.; Claudia Neunaber, Prof. Dr. rer. nat; Arndt F. Schilling, Prof. Dr. med.; Christian Mühlfeld, Prof. Dr. med.; Stephan Sehmisch, Prof. Dr. med.; Tilman Graulich, PD Dr. med.

Corresponding author:

**Tilman Graulich, PD Dr. med.**

Hannover Medical School, Department of Trauma Surgery

Carl-Neuberg Str. 1, Hannover D-30625, Germany

E-mail: [graulich.tilman@mh-hannover.de](mailto:graulich.tilman@mh-hannover.de)

Mobil: 004917615326723

Tel.: 00495115322050

ORCID: 0000-0003-1896-1945


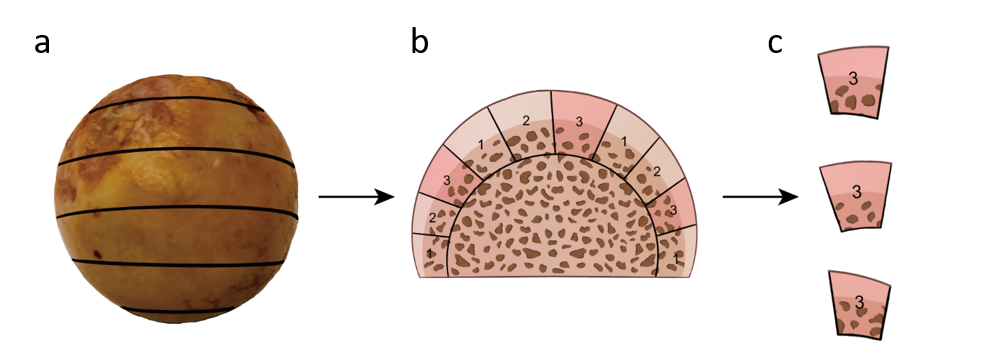


Fig. S1: The procedures of cutting femoral head. (A) The femoral head was divided to several slices with 1 cm width. (B) The femoral head slice was divided to 1 cm × 1 cm × 1 cm cubes from the outer cartilage. (C) The certain femoral heads cubes were selected to next experiments.


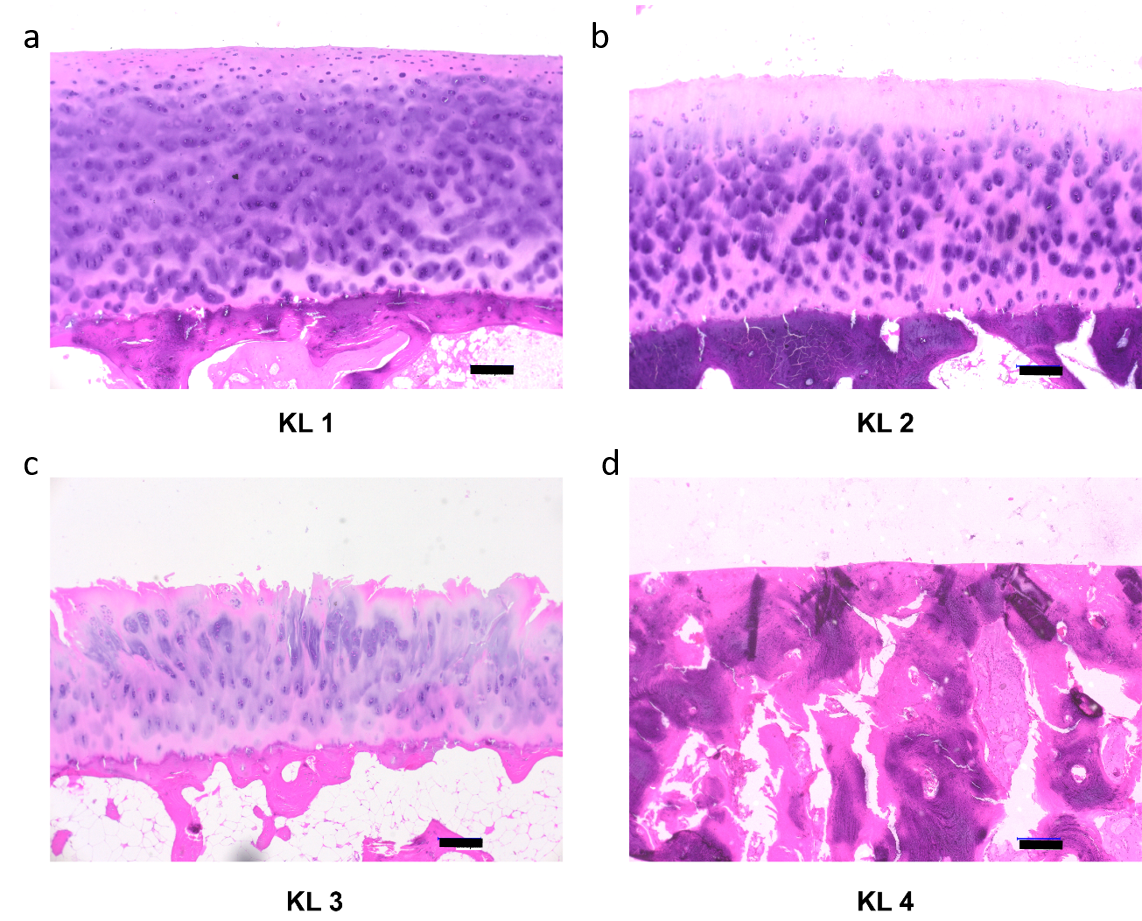


Fig S2. Cartilage thickness analysis with OA progression. (A – D) The varying cartilage thickness across groups KL 1 – 4. (A) The cartilage appears relatively thick and well-preserved in group KL 1. (B) The cartilage retains significant thickness in group KL 2. (C) In group KL 3, the cartilage exhibits slightly decreased thickness. (D) In group KL 4, the cartilage is completely absent. Scale bar: 250 μm. N_patients_ = 43 (total femoral heads), n_biopsies_ = 129 (3 biopsies/femoral head). Scale bar: 250 µm.

Table S1. Correlation of vessel values and Mankin scores

|  | *rs* (Vessel values) | R^2^ | *p* |
| --- | --- | --- | --- |
| Volume | 0.603 | 0.302 | <0.001 |
| Surface Area | 0.563 | 0.281 | <0.001 |
| Length | 0.315 | 0.009 | <0.001 |

Table S2. Stereological analysis of cartilage and subchondral bone changes.

| Group | | KL 1 | KL 2 | KL 3 | KL 4 | Total | *p* |
| --- | --- | --- | --- | --- | --- | --- | --- |
| n | | 6 | 14 | 10 | 13 | 43 | *-* |
| Mankin Score | | 1 (IQR: 0 – 4.25) * | 4 (IQR: 1 - 5) * | 3 (IQR: 1 - 6) * | 14 (IQR: 6 - 14) | 4 (IQR: 1 - 8.5) | < 0.001 |
|  | | | | | | | |
| Cartilage | |  | | | | | |
| Thickness (mm) | | 1.60 (IQR: 1.33 – 1.94) * | 1.72 (IQR: 1.13 - 2.13) * | 1.83 (IQR: 1.50 - 2.30) * | 0 (IQR: 0 - 1.49) | 1.55 (IQR:0.90 - 2.06) | < 0.001 |
| Chondrocytes | Vv (Chondrocytes/Cartilage) (%) | 6.99 (IQR: 6.06 – 8.14) | 6.52 (IQR: 4.89 – 8.49) | 7.58 ± 2.27 | 6.20 ± 1.75 | 6.77 (IQR: 5.63 - 8.37) | 0.283 |
|  | V (Chondrocytes, Cartilage) (cm^3^) | 1.05 ± 0.34 * | 1.16 ± 0.42 * | 1.19 ± 0.43 * | 0.71 ± 0.16 | 1.06 ± 0.40 | < 0.001 |
| ECM | Vv (ECM/Cartilage) (%) | 93.01 (IQR: 91.86 – 93.94) | 93.48 (IQR: 91.51 – 95.11) | 92.42 ± 2.27 | 93.66 ± 1.72 | 93.19 (IQR: 91.62 – 94.35) |  |
|  | V (ECM, Cartilage) (cm^3^) | 12.57 ± 2.59 * | 14.58 ± 3.49 * | 14.05 ± 3.68 * | 11.28 ± 3.02 | 13.49 ± 3.44 | < 0.001 |
|  | | | | | | | |
| Subchondral region | |  | | | | | |
| Subchondral bone | Vv (Subchondral bone/Subchondral region) (%) | 50.77 ± 7.97 * | 55.67 ± 8.55 * | 50.76 ± 6.40 * | 71.79 ± 14.05 | 55.00 (IQR: 49.34 - 66.67) | < 0.001 |
|  | V (Subchondral bone, Subchondral region) (cm^3^) | 2.43 ± 0.29 * | 2.89 ± 0.73 * | 2.70 ± 0.55 * | 4.08 ± 1.66 | 2.70 (IQR: 2.37 - 3.74) | < 0.001 |
| Bone marrow | Vv(Bone marrow /Subchondral region) (%) | 49.23 ± 7.97 * | 44.33 ± 8.55 * | 49.24 ± 6.40 * | 28.20 ± 14.05 | 45.00 (IQR:33.33 - 50.66) | < 0.001 |
|  | V (Bone marrow, Subchondral region) (cm3) | 2.38 ± 0.54 * | 2.32 ± 0.80 * | 2.60 ± 0.43 * | 1.54 ± 0.70 | 2.16 ± 0.77 | < 0.001 |

*: *p*<0.05 vs. Group KL4

Table S3. Correlation of cartilage and subchondral region values and Mankin scores

|  | *rs* (Vessel values) | *R^2^* | *p* |
| --- | --- | --- | --- |
| Cartilage | -0.738 | 0.667 | <0.001 |
| Chondrocytes | -0.223 | 0.299 | 0.023 |
| ECM | 0.223 | 0.299 | 0.023 |
| Subchondral bone | 0.434 | 0.384 | <0.001 |
| Bone marrow | -0.434 | 0.384 | <0.001 |
